# Prediction models for sarcopenia risk in dialysis patients: a systematic review and critical appraisal

**DOI:** 10.1007/s40520-024-02911-7

**Published:** 2025-01-03

**Authors:** Zhuoer Hou, Xiaoyan Li, Lili Yang, Ting Liu, Hangpeng Lv, Qiuhua Sun

**Affiliations:** 1 The College of Nursing, Zhejiang Chinese Medical University, Hangzhou, China; 2 The Affiliated Shaoyifu Hospital of Zhejiang University, Hangzhou, China; 3 The College of Basic Medicine, Zhejiang Chinese Medical University, Hangzhou, China; 4 Department of stomatology, Haining Traditional Chinese Medicine Hospital, Jiaxing, China

**Keywords:** Sarcopenia, Dialysis, Prediction models, Systematic review, Critical appraisal

## Abstract

**Background:**

Many studies have developed or validated predictive models to estimate the risk of sarcopenia in dialysis patients, but the quality of model development and the applicability of the models remain unclear.

**Objective:**

To systematically review and critically evaluate currently available predictive models for sarcopenia in dialysis patients.

**Methods:**

We systematically searched five databases until March 2024. Observational studies that developed or validated predictive models or scoring systems for sarcopenia in dialysis patients were considered eligible. We included studies of adults (≥ 18 years of age) on dialysis and excluded studies that did not validate the predictive model. Data extraction was performed independently by two authors using a standardized data extraction table based on a checklist of key assessments and data extraction for systematic evaluation of predictive modeling research. The quality of the model was assessed using the Predictive Model Risk of Bias Assessment Tool.

**Results:**

Of the 104,454 studies screened, 13 studies described 13 predictive models. The incidence of sarcopenia in dialysis patients ranged from 6.6 to 34.4%. The most commonly used predictors were age and body mass index. In the derivation set, the reported area under the curve or C-statistic is between 0.81 and 0.95. The area under the curve reported by the external validation set is between 0.78 and 0.93. All studies had a high risk of bias, mainly due to poor reporting in the outcome and the analysis domains, and three studies had a high risk of bias in terms of applicability.

**Conclusion:**

Future research should focus on validating and improving existing predictive models or developing new models using rigorous methods.

**Supplementary Information:**

The online version contains supplementary material available at 10.1007/s40520-024-02911-7.

## Introduction

As a geriatric syndrome, sarcopenia is a geriatric disease in which the quantity, quality, and physical function of skeletal muscle decrease and decline with age [1]. The onset of sarcopenia is often hidden. It frequently leads to serious adverse consequences such as body dysfunction, falls, disability, increased length of hospitalization, and increased mortality, which seriously damages the quality of life and health of the elderly [1–3]. However, recent studies have shown that sarcopenia is not only related to age, but is also strongly associated with many diseases, such as cancer, diabetes, chronic obstructive pulmonary disease (COPD), and organ failure [4]. Among them, sarcopenia occurs early and develops rapidly in patients undergoing dialysis due to renal failure, and its prevalence rate is between 3.9% and 63.3% [5]. The development of sarcopenia is exacerbated by the acceleration of protein catabolism during the disease and dialysis, which leads to an increase in inflammatory factors, electrolyte imbalances, and hormonal imbalances. Additionally, anorexia, low energy intake, and reduced protein consumption further contribute to the progression of this condition [6, 7].

Prevention is currently the primary option for dialysis patients with sarcopenia. Due to the complexity of its pathogenesis, the exact mechanism remains unclear, and there is no targeted treatment available [8]. Furthermore, the current clinical diagnostic methods for sarcopenia are characterized by high measurement costs, time-consuming procedures, potential radiation hazards, and operational complexity. These factors impede the early identification of high-risk patients with sarcopenia by medical staff [9]. The simple and user-friendly predictive model can assist healthcare professionals in screening high-risk groups and aid them in implementing appropriate preventive measures based on different risk stratification to optimize resource utilization.

In recent years, an increasing amount of research has been dedicated to developing or validating predictive models for sarcopenia in dialysis patients. However, the quality and applicability of model development remain uncertain. Medical staff often lack clarity on which model to utilize and which populations and settings the model pertains to. Consequently, we conducted a systematic review and critical evaluation of all currently available predictive models for sarcopenia in dialysis patients to inform further research in this domain.

## Methods

### Study design

This systematic review was registered in PROSPERO before initiation of the search (Registration ID: CRD42024520767).

### Data sources and eligibility criteria

**Fig. 1:**
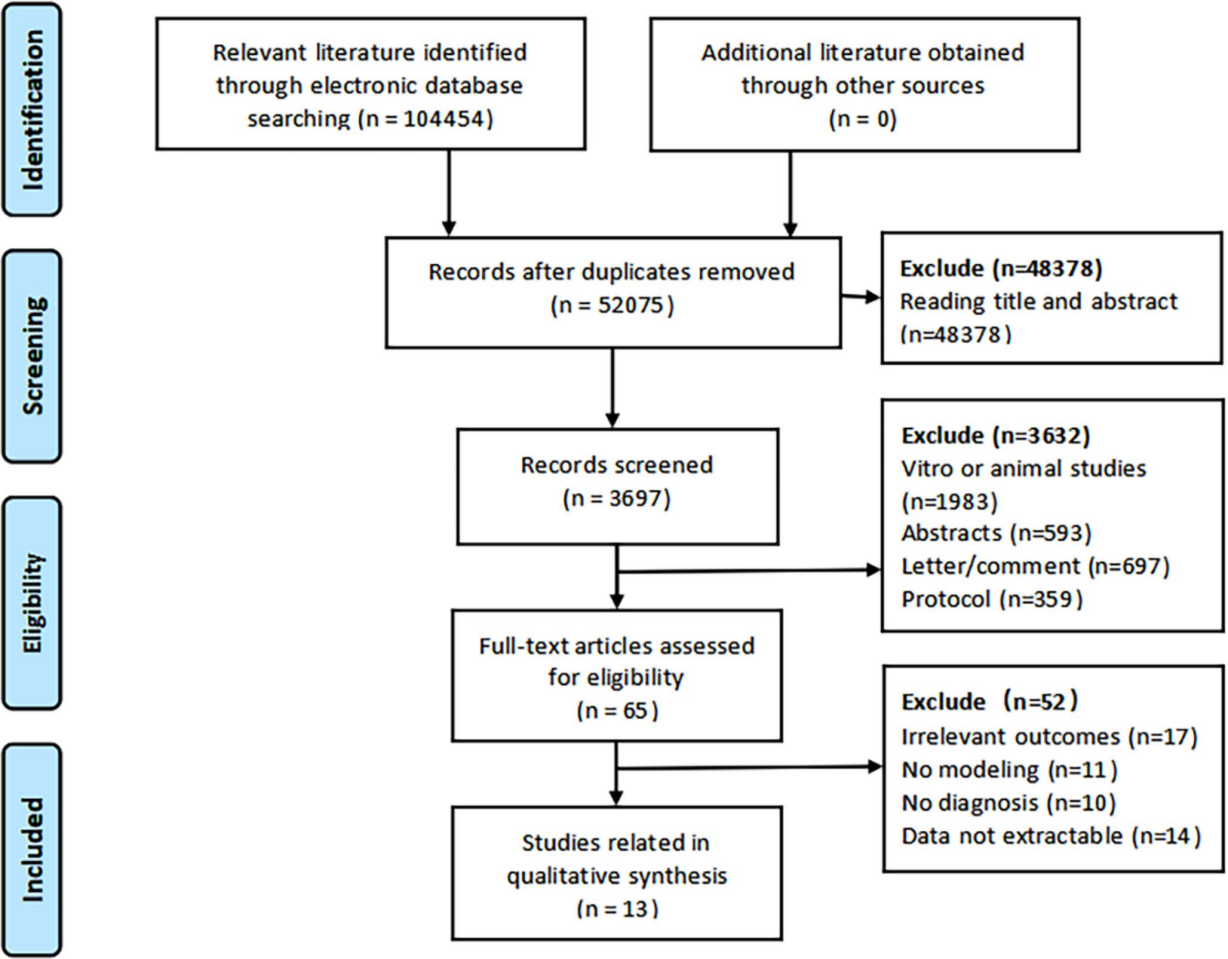
Flow diagram of literature selecting process and results according to the preferred reporting items for systematic reviews and meta-analysis (PRISMA)

Two researchers independently searched the following English and Chinese electronic databases from inception to March 2024: PubMed, Web of Science (WOS), Embase, the Cochrane Library and China National Knowledge Internet (CNKI). The following keywords were used to conduct a basic search: “sarcopenia”, “sarcopenic”, “muscle mass”, “muscle strength”, “hand strength”, “grip strength”, “muscle atrophy”, “muscle wasting”, “prediction model”, “prediction”, “predict model”, “risk prediction”, “risk factors”, “risk assessment”, “prognostic model”, “model”, “nomogram”. (Specific details regarding the strategies are in the Supplemental materials.) All references included in this review and references from previous relevant systematic reviews were also checked for any additional studies. Figure 1 shows the process of screening articles.

Observational studies were considered eligible if they developed or validated prediction models or scoring systems for the occurrence of sarcopenia in dialysis patients. We used the PICOTS system that was recommended in the Critical Appraisal and Data Extraction for Systematic Reviews of Prediction Modeling Studies (CHARMS) checklist [10] to describe the key items for our systematic review as follows.

P (Population): The population of interest comprises patients undergoing dialysis who are aged over 18 years old.

I (Intervention model): Studies focus on prediction models that have been internally or externally validated.

C (Comparator): None.

O (Outcome): Sarcopenia. The definition of sarcopenia is in line with the internationally recognized consensus of Asian Working Group on Sarcopenia (AWGS) [9], European Working Group on Sarcopenia in Older People (EWGSOP) [11], International Working Group on Sarcopenia (IWGS) [12] and National Foundation for Health Research (FNIH) [13].

T (Timing): Outcomes were predicted using post-dialysis conditions.

S (Setting): The intended use of the prediction model was to perform risk stratification in the assessment of sarcopenia development in dialysis, enabling the implementation of preventive measures.

### Study selection and screening

The two researchers selected the literature independently. Duplicates were removed using Endnote X9 software. The first filter was then made by reading the title and summary. Finally, read the full text of the remaining articles was read for a second screening to determine the final inclusion of each article. The reasons for excluding each article from the first and second screenings were recorded. Any discrepancies were resolved by a third researcher.

### Data extraction and quality assessment

The data extraction was conducted independently by two authors using a standardized data extraction table based on the CHARMS checklist [10]. Data items such as study characteristics, outcome measures, predictors and performance were extracted. The predictive performance of the models was extracted by using any measures proposed in the study.

The study quality was independently assessed by two authors using PROBAST (Predictive Model Risk of Bias Assessment Tool). PROBAST aims to evaluate preliminary studies that develop, validate or update multivariate predictive models for diagnosis or prediction [14]. The focus of this review is on diagnostic prediction models to predict the probability of developing sarcopenia in dialysis patients. PROBAST consists of four domains containing 20 signaling questions for the risk of bias and applicability assessment. The four domains are as follows: (1) Participants: the sources of data and criteria for inclusion and exclusion of subjects; (2) Predictors: the definition of predictors and its measurement method, process and time point; (3) Outcome: the definition of outcome and its measurement method, process and time point; (4) Analysis: whether the main statistical factors are correctly treated. Signal questions are factual and can be answered as “yes” (Y), “probably” (PY), “no” (N), “probably not” (PN), or “no information” (NI). The overall risk of bias and concerns about the applicability of the predictive model were judged to be low, high, or unclear [14]. Any discrepancies were resolved by a third researcher.

### Data synthesis

A descriptive analysis of the included studies was conducted in table form to summarize the main features of the predictive models. Data in the table included general information about the included studies, participants, age, main outcome, diagnostic criteria, assessment method, sample size, predictors included, statistical methods, and predictive performance. The predictive performance of the prediction models for sarcopenia risk in dialysis patients is measured by discrimination (area under the curve or C-statistic) and calibration (calibration curve or Hosmer-Lemeshow test). Some studies could report on the sensitivity, specificity and clinical validity (decision curve analysis) of predictive models. Due to the heterogeneity of the predictors and the characteristics of the participants included in the prediction models, all results were summarized and described descriptively without any quantitative synthesis.

## Result

We retrieved 104,454 records through a systematic search. After removing the duplicate studies, the titles and abstracts of 3,697 articles were read for eligibility screening, of which 65 met the eligibility criteria. Next, upon reading the full text for screening, we excluded 17 studies with outcomes other than sarcopenia, 11 studies that were not modeled, 10 studies that did not provide diagnostic criteria for sarcopenia, 14 studies for which data could not be extracted, and 13 studies that were included in our systematic review [15–27].

### Description of included models

#### Characteristics of model derivation

From the included studies, we identified 13 models that predicted the risk of sarcopenia in dialysis patients. Table 1 provides an overview of the development and performance of the included models. The reported incidence of sarcopenia in dialysis patients ranged from 6.6 to 34.4%. One model was for peritoneal dialysis patients [27] and 12 models were for maintenance hemodialysis patients [15–26]. Eight studies were cross-sectional [15, 16, 20–24, 27], two were prospective cohort studies [17, 26], and three studies used retrospective data to establish predictive models for sarcopenia in dialysis patients [18, 19, 25]. Sample sizes ranged from 105 to 589. The number of predictors for these models ranged from 3 to 12. Logistic regression analysis was used to establish predictive models for all included studies. Both bioelectrical impedance analyzer (BIA) and dual energy X-ray absorptiometry (DXA) are commonly used to screen for sarcopenia, while computed tomography (CT) was used in only one model.

**Table 1:** Overview of model development for included prediction models

Author (year), country	Study design	Participants	Age^a^	Main outcome	Diagnostic criteria	Assessment method	Sarcopenia cases/ sample size(%)	Predictors in final model	Performance^b^
Lin (2020), China	Cross-section	Aged 20 years and older, MHD	59.2 ± 11.9	Sarcopenia	AWGS	DXA	14/214 (6.6%)	Total body water, body weight, gender, age	AUC: 0.945 (men), 0.940 (women) Sensitivity: 94.1% Specificity: 98.8% PPV: 84.2% NPV: 99.6%
Du (2021), China	Cross-section	Aged 18 years and older, MHD	54.11 ± 14.45	Sarcopenia	AWGS	BIA	74/304 (24.3%)	Gender, BMI, calf circumference, predialysis creatinine	AUC: 0.911 (95%CI 0.873–0.940) Sensitivity: 0.919 Specificity: 0.783 Accuracy: 75.40%
Xie (2022), China	Cross-section	Aged 18 years and older, MHD	60.0 ± 12.5	Sarcopenia	AWGS	BIA	77/511 (15.1%)	Age, sex, body weight, grip strength	Youden index: 0.725 C-index: 0.929 (95% CI: 0.904–0.953) Sensitivity: 0.909 Specificity: 0.816
Ding (2022), China	Cross-section	Aged 18 years and older, MHD	59.19 ± 13.59	Sarcopenia	AWGS	BIA	-/206 (-%)	Age, BMI, phase angle	AUC: 0.925 (95%CI 0.892–0.958) Sensitivity: 86.8% Specificity: 79.2%
Wu (2022), China	Cross-section	Aged 18–65 years, PD	54.2 ± 8.89	Sarcopenia	AWGS	BIA	33/105 (31.4%)	Grip strength, BMI, total body water value, irisin, extracellular water, fat-free mass index, phase angle, albumin/globulin, blood phosphorus, total cholesterol, triglyceride, prealbumin	AUC: 0.82 (95%CI 0.67-1.00) Specificity: 0.96 Sensitivity: 0.91 PPV: 0.96 NPV: 0.91 AUC: 0.84 (95%CI 0.61–0.95), Specificity: 0.92 Sensitivity: 0.73 PPV: 0.90 NPV: 0.77
Bao (2022), China	Retrospective cohort	Aged 18 years and older, MHD	67.14 ± 4.08	Sarcopenia	AWGS	BIA	56/206 (27.2%)	Age, cognitive dysfunction, Hcy, malnutrition-inflammation score, Irisin	AUC: 0.889 Sensitivity: 94.23% Specificity: 67.33% AUC: 0.894 Sensitivity: 95.44% Specificity: 71.22% AUC: 0.835 Sensitivity: 91.33% Specificity: 76.34%
Qin (2022), China	Retrospective cohort	Aged 18 years and older, MHD	Age ≥ 60 years (87.68%)	Sarcopenia	AWGS	BIA	73/318 (22.96%)	Age, severe malnutrition, hypersensitive C-reactive protein, moderate and severe physical activity	AUC: 0.83 (95%CI: 0.78–0.88) DCA: 0.08–0.98
Zhou (2022), China	Cross-section	Aged 18–60 years, MHD	46.00 (37.00, 53.50)	Sarcopenia	AWGS	BIA	41/237 (17.3%)	BMI, upper arm muscle circumference, hemoglobin	AUC: 0.862 (95%CI 0.792–0.932)
Du (2022), China	Cross-section	Aged 18 years and older, MHD	54 (44, 64)	Sarcopenia	AWGS	BIA	101/589 (17.1%)	Age, BMI, calf circumference, serum creatinine	AUC: 0.922 (95%CI 0.899–0.946) Sensitivity: 85.1% Specificity: 85.9% Youden index: 0.710
Cai (2022), China	Retrospective cohort	Aged 18 years and older, MHD	Age ≥ 60 years (47.7%)	Sarcopenia	AWGS	BIA	59/369 (16.0%)	Age, C-reactive protein, serum phosphorus, BMI, mid-upper arm muscle circumference	AUC: 0.869 (95%CI 0.822–0.915) Sensitivity: 77% Specificity: 83% Youden index: 0.60
Qin (2023), China	Prospective cohort	MHD	58.92 ± 13.39	Sarcopenia	AWGS	BIA	58/246 (23.6%)	Age, years of haemodialysis, nutrition status score	C-index: 0.919 (95%CI 0.340–0.939) AUC: 0.924 Sensitivity: 91.30% Specificity: 77.38% Youden index: 0.687
Senzaki (2023), Japan	Prospective cohort	MHD	70.2 ± 11.4	low muscle mass	Psoas muscle mass index	CT	150/441 (34.1%)	Grip strength, sex, height, dry weight, primary cause of end-stage renal disease, diastolic blood pressure at start of session, pre-dialysis potassium and albumin level, dialysis water removal in a session	AUC: 0.81 Sensitivity: 60% Specificity: 87%
Tian (2023), China	Cross-section	Aged 18 years and older, MHD	66.81 ± 12.95	low muscle mass	AWGS	BIA	84/244(34.4%)	Age, sex, BMI, handgrip strength, gait speed	AUC: 0.906 (95%CI 0.862–0.940) Sensitivity: 85.71% Specificity: 80.62%

MHD = maintenance hemodialysis, BIA = bioelectrical impedance analyzer, BMI = body mass index, DXA = dual X-ray absorptiometry, AUC = area under the curve, CI = condence interval, PD = peritoneal dialysis, SD = standard deviation, IQR = interquartile rage, AWGS = Asia Working Group for Sarcopenia criteria, PPV = positive predictive value, NPV = negative predictive value, ROC = receiver operator characteristic curve, CT = computed tomography, DCA = decision curve analysis

^a^ Age are showed in mean ± SD, median (IQR, range) or percentage according to the reported data

^b^ C index, sensitivity (%), specicity (%), PPV/NPV (%), calibration slope, other (95%CI, if reported). Generally, we consider AUC = 0.5–0.7 as poor discrimination, 0.7–0.8 as moderate discrimination, 0.8–0.9 as good discrimination, 0.9-1.0 as excellent discrimination

#### Included predictors

**Fig. 2:**
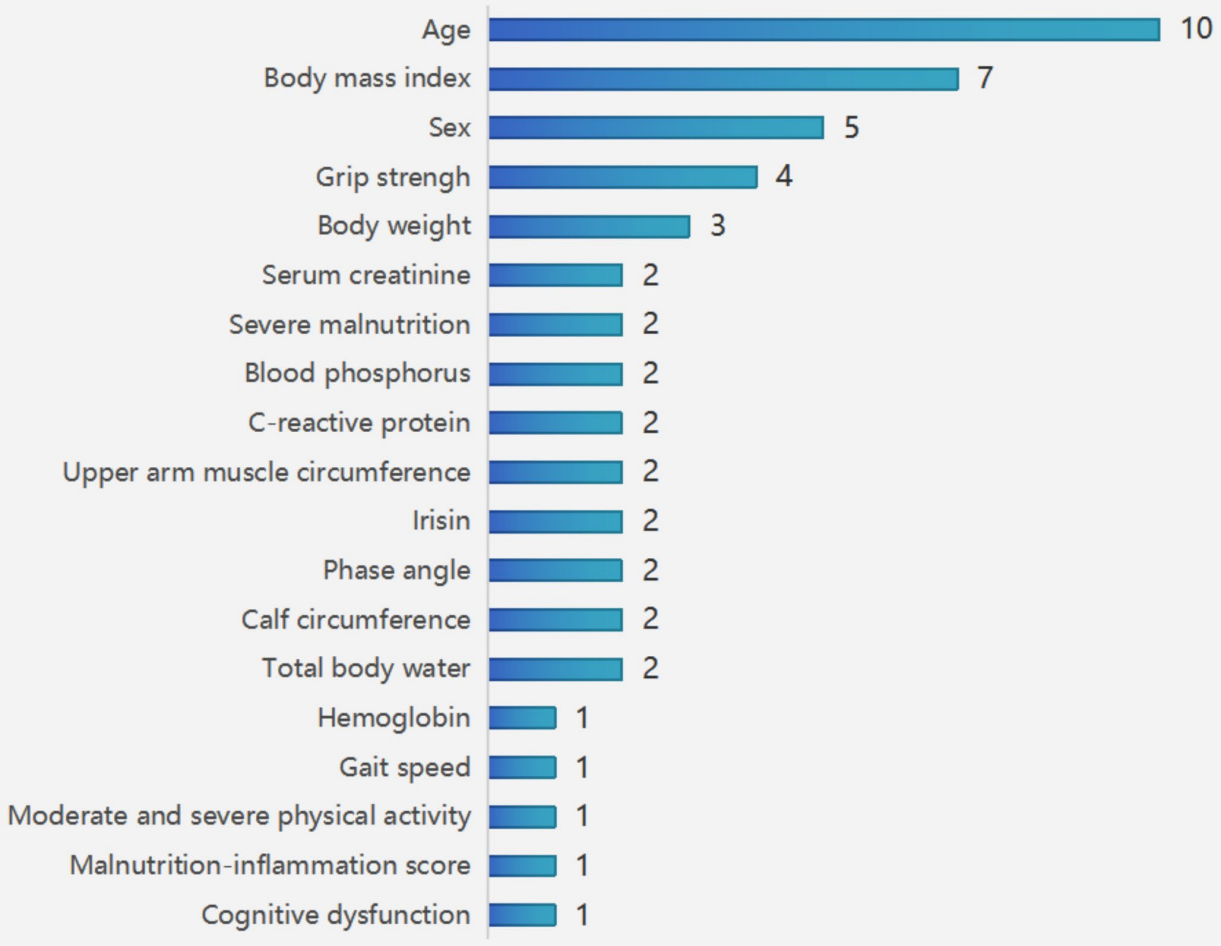
Summary of predictors present in included models

The most commonly used predictors were age, body mass index (BMI), sex, grip strength, and weight, which appeared in 10 models, 7 models, 5 models, 4 models, and 3 models, respectively. Other commonly used predictors included total body water, calf circumference, phase angle, irisin, upper arm muscle circumference, C-reactive protein, blood phosphorus, severe malnutrition and serum creatinine, which were used twice (Fig. 2).

#### Model performance in the derivation set

In the derivation set, each of the 13 models reported areas under the curve (AUCs) or C-statistics for the model, with a reporting range of 0.81 to 0.95. The calibration of 10 models was reported, 6 studies were presented as calibration curves, and 4 studies were presented as Hosmer-Lemeshow (H-L) good-of-fit tests, all of which showed good performance.

### Model validation

Table 2 shows an overview of model validation for the included prediction models. Eight models were only internally validated [15, 17–20, 23, 25, 27], and three models were only externally validated [16, 22, 24]. Xie’s model and Senzaki’s model were validated both internally and externally [21, 26]. The AUCs or C-statistics for internal validation reports is between 0.78 and 0.93. The reporting range for external validation is 0.73 to 0.96.

**Table 2:** Overview of model validation for included prediction models

Model	Citation of validation (year)	Type of validation	Participants	Age^a^	Diagnostic criteria	Assessment method	Sarcopenia cases/ sample size(%)	Performance^b^
Lin’s model (2020)	Lin (2020)	External validation	Aged 20 years and older, MHD	61.0 ± 10.7	AWGS	DXA	8/108 (7.3%)	AUC: 0.945 (men), 0.940 (women) Sensitivity: 94.1% Specificity: 98.8% PPV: 84.2% NPV: 99.6%
Du’s model (2021)	Du (2021)	External validation	Aged 18 years and older, MHD	54.08 ± 13.82	AWGS	BIA	68/309 (22.0%)	AUC: 0.819 Sensitivity: 0.800 Specificity: 0.737 Accuracy: 75.40%
Xie’s model (2022)	Xie (2022)	Internal validation	Aged 18 years and older, MHD	61.2 ± 11.3	AWGS	BIA	77/511 (15.1%)	C-index: 0.929 (95% CI 0.904–0.953)
Xie’s model (2022)	Xie (2022)	External validation	Aged 18 years and older, MHD	61.2 ± 11.3	AWGS	BIA	47/246 (19.1%)	C-index: 0.955 (95% CI 0.931–0.979)
Ding’s model (2022)	Ding (2022)	Internal validation	Aged 18 years and older, MHD	56.68 ± 13.94	AWGS	BIA	32/140 (22.9%)	AUC: 0.917 (95%CI 0.872–0.961) Sensitivity: 89.2% Specificity: 81.6%
Wu’s model (2022)	Wu (2022)	Internal validation	Aged 18–65 years, PD	54.2 ± 8.89	AWGS	BIA	33/105 (31.4%)	AUC: 0.82 (95%CI 0.67-1.00) Specificity: 0.96 Sensitivity: 0.91 PPV: 0.96 NPV: 0.91 AUC: 0.84 (95%CI 0.61–0.95), Specificity: 0.92 Sensitivity: 0.73 PPV: 0.90 NPV: 0.77
Bao’s model (2022)	Bao (2022)	Internal validation	Aged 18 years and older, MHD	67.14 ± 4.08	AWGS	BIA	56/206 (27.2%)	C-index: 0.822 (95%CI 0.734–0.887) AUC: 0.829 (95%CI: 0.742–0.916)
Qin’s model (2022)	Qin (2022)	Internal validation	Aged 18 years and older, MHD	Age ≥ 60 (87.68%)	AWGS	BIA	73/318 (22.96%)	AUC: 0.83 (95%CI: 0.78–0.88) DCA: 0.08–0.98
Zhou’s model (2022)	Zhou (2022)	Internal validation	Aged 18–60 years, MHD	46.00 (37.00, 53.50)	AWGS	BIA	-/102 (-%)	AUC: 0.866 (95%CI: 0.788–0.943) Sensitivity: 89.5% Specificity: 74.7% Youden index: 0.642
Du’s model (2022)	Du (2022)	External validation	Aged 18 years and older, MHD	54 (44, 64)	AWGS	BIA	35/216 (16.2%)	Accuracy: 83.8% AUC: 0.913 (95%CI 0.870–0.956) Sensitivity: 94.3% Specificity: 82.9% Youden index: 0.772
Cai’s model (2022)	Cai (2022)	Internal validation	Aged 18 years and older, MHD	Age ≥ 60 years (51.2%)	AWGS	BIA	43/246 (17.5%)	AUC: 0.832 (95%CI 0.765 -0.900) Sensitivity: 70% Specificity: 88% Youden index: 0.58 C-index: 0.783 Accuracy: 82%
Qin’s model (2023)	Qin (2023)	Internal validation	MHD	58.92 ± 13.39	AWGS	BIA	58/246 (23.6%)	C-index: 0.919 (95%CI 0.340–0.939) AUC: 0.924 Sensitivity: 91.30% Specificity: 77.38% Youden index: 0.687
Senzaki’s model (2023)	Senzaki (2023)	Internal validation	MHD	70.2 ± 11.4	Psoas muscle mass index	CT	150/441 (34.1%)	AUC: 0.81 Sensitivity: 60% Specificity: 87%
Senzaki’s model (2023)	Senzaki (2023)	External validation	MHD	67.2 ± 12.7	Psoas muscle mass index	CT	70/178 (39.3%)	AUC: 0.73 Sensitivity: 64% Specificity: 82%
Tian’s model (2023)	Tian (2023)	Internal validation	Aged 18 years and older, MHD	66.02 ± 9.18	AWGS	BIA	45/102 (44.1%)	AUC: 0.917 (95%CI 0.846–0.962) Sensitivity: 80.00% Specificity: 80.70%

MHD = maintenance hemodialysis, BIA = bioelectrical impedance analyzer, DXA = dual X-ray absorptiometry, AUC = area under the curve, CI = condence interval, PD = peritoneal dialysis, SD = standard deviation, IQR = interquartile rage, AWGS = Asia Working Group for Sarcopenia criteria, PPV = positive predictive value, NPV = negative predictive value, ROC = receiver operator characteristic curve, CT = computed tomography, DCA = decision curve analysis

^a^ Age are showed in mean ± SD, median (IQR, range) or percentage according to the reported data

^b^ C-index, sensitivity (%), specicity (%), PPV/NPV (%), calibration slope, other (95%CI, if reported). Generally, we consider AUC = 0.5–0.7 as poor discrimination, 0.7–0.8 as moderate discrimination, 0.8–0.9 as good discrimination, 0.9-1.0 as excellent discrimination

### Risk of bias and applicability

The overall and domain-specific results for the risk of bias and applicability of the 13 included studies are shown in Table 3. Twelve studies had a high risk of bias [16–27], while one study had unclear risks [15], suggesting that there were some issues during model development or validation. In the participant domain, three studies exhibited a high risk of bias, mainly due to retrospective design [18, 19, 25]. In the predictor domain, three studies had a high risk of bias [18, 19, 25]. This was mainly due to the retrospective design, as the measurement of predictors after the occurrence of outcomes is susceptible to interference from the occurrence of outcomes during the measurement process. The risk of bias is substantial, and the quality of the assessment of predictors cannot be adequately controlled. In the outcome domain, six studies had a high risk of bias. Of these, three studies reported the inclusion of predictors in the definition of outcomes [21, 23, 27]. One study used predictor information in determining outcomes [27]. Furthermore, due to the retrospective nature of the three studies, the information of predictors was clear when determining the results, and the quality of the outcome assessment could not be controlled [18, 19, 25]. In the analysis domain, twelve studies had a high risk of bias, while one study remained unclear. The ratio of the number of subjects to the number of candidate predictors for the outcome of eleven studies was less than 20 [16–23, 25–27]. The “events per variable” (EPVs) could not be calculated in the model development study of one study [15]. Two studies converted continuous variables into categorical variables without a clear classification basis [18, 24]. No information on continuous or categorical variables was reported in the two studies [15, 27]. In three studies, the missing participants were directly excluded [20, 26, 27]. The participants of the three studies were excluded by missing data, without clarifying how to address the missing data [17, 20, 27]. Three studies screened the predictors based on single factor analysis, which could lead to the omission of independent variables and lead to bias [16, 18, 20]. Eleven studies did not provide any information on whether there was complexity in the data [15–21, 23–25, 27]. The internal validation of one study consisted only of random split validation of the data and did not assess subsequent adjustments to the model’s performance [22].

**Table 3:** PROBAST results of included studies

Study Author (year)	Study type	ROB				Applicability			Overall	
		Participants	Predictors	Outcome	Analysis	Participants	Predictors	Outcome	ROB	Applicability
1-Lin (2020)	B	+	+	+	-	+	+	+	-	+
2-Du (2021)	B	+	+	+	-	+	+	+	-	+
3-Xie (2022)	B	+	+	-	-	+	+	+	-	+
4-Ding (2022)	A	+	+	+	?	+	+	+	?	+
5-Wu (2022)	A	+	+	-	-	+	+	+	-	+
6-Bao (2022)	A	-	-	-	-	+	+	+	-	+
7-Qin (2022)	A	-	-	-	-	+	+	+	-	+
8-Zhou (2022)	A	+	+	+	-	-	+	+	-	-
9-Du (2022)	B	+	+	+	-	+	+	+	-	+
10-Cai (2022)	A	-	-	-	-	+	+	+	-	+
11-Qin (2023)	A	+	+	+	-	+	+	+	-	+
12-Senzaki (2023)	B	+	+	+	-	+	+	-	-	-
13-Tian (2023)	A	+	+	-	-	+	+	-	-	-

PROBAST = Prediction model Risk Of Bias Assessment Tool; ROB = risk of bias

A indicates“development only”; B indicates “development and validation in the same publication”; C indicates “validation only”

+ indicates low ROB/low concern regarding applicability; − indicates high ROB/high concern regarding applicability; and? indicates unclear

ROB/unclear concern regarding applicability

Overall, 3 studies had a high risk of applicability [20, 23, 26]. With regard to the participant domain of applicability, one study had a high risk, mainly because the study did not focus on dialysis patients with sarcopenia of all ages. Regarding the predictor domain, the overall risk is low. Regarding the outcome domain, the definitions, methods, and timing of the original findings of the two studies were inconsistent with the questions of the systematic review. The outcome of both studies was low muscle mass, and one of the studies was diagnosed by computed CT and psoas muscle mass index.

## Discussion

Of the 13 studies included, 12 had a high risk of bias, and 1 had an unclear risk. Three studies were considered to have high concern regarding applicability according to the PROBAST. The main reasons include: some data came from retrospective studies, insufficient samples of positive events, improper conversion of continuous variables and categorical variables, improper processing of missing data, improper selection of predictors, failure to consider the complexity of the data, lack of external validation of the model, and failure to consider the overfitting of the model. Internal validation of the prediction model is used to check the repeatability of the model to prevent overfitting [28], while external validation focuses on the portability and generalization of the model [29]. Of the articles included in this study, 12 were from China, all published in the last three years. It can be observed that research on risk prediction models for dialysis patients with sarcopenia has increased rapidly, and attention has been paid to the performance and verification of the testing models. Most models are presented in the form of a nomogram, which is more intuitive and convenient, providing patients with accurate and personalized risk predictions, thereby facilitating clinicians to effectively screening high-risk patients and taking timely intervention measures. The availability of rigorous predictive models is limited, and more high-quality research is needed to advance this field.

The reported incidence varies widely, mainly due to the age of the target population included in the study. The incidence of sarcopenia is higher in elderly patients. The occurrence of sarcopenia is strongly associated with low BMI and can therefore occur in patients of any age, making it necessary to screen patients of all ages. The occurrence of sarcopenia is strongly associated with low BMI and can therefore occur in patients of any age, making it necessary to screen patients of all ages [30]. Eleven studies focused on dialysis patients of every age, and two studies focused on populations that did not include the elderly. Because sarcopenia results from multiple influences, the predictors in the models of our systematic review vary. In these models, age was the most common predictor identified as a risk factor, supported by strong evidence. Some laboratory test indicators, such as body moisture, irisin, phase angle, C-reactive protein, blood phosphorus, and serum creatinine, require professional instruments and expertise to ensure the accuracy of the evaluation results. It is noteworthy that grip strength, body weight, calf circumference, and upper arm circumference have been identified in some studies as predictors of sarcopenia in dialysis patients [20, 22, 24, 27]. These factors are the mutual causes of sarcopenia. Specifically, grip strength, body weight, calf circumference, and upper arm circumference can promote the development of sarcopenia, but sarcopenia may also lead to low grip strength, low body weight, and reduced calf circumference. Therefore, the definitions of these predictors and the time points at which they are assessed should be clearly described. From the performance of these models, most demonstrate good discriminative ability in their respective external validation data. It is recommended that performance be tested in additional studies.

PROBAST was developed and published in 2019 [14], and the articles included in this study are all from after 2019. However, when we critically evaluated the included studies according to the PROBAST criteria, all studies were rated as having a high risk of bias, mainly due to poor reporting of outcomes and analysis domains. First, although most of the studies had a prospective design, three of them were retrospective. This means that these studies did not take into account the blinding of the outcome determination and prediction information. The predictive factors and outcome indicators of the research object should adopt the same definition and the same measurement method, and the measurement should adopt the blind method and select the appropriate time point. Secondly, the small sample size of the included literature is also a common problem. The incidence of sarcopenia is not very high, there are many candidate predictors, and if the number of events per variable (EPV) is < 10, then overfitting may occur [31, 32]. This means that the performance of these models may be affected by the researchers’ overestimation. Furthermore, the transformation of continuous variables into categorical variables should be avoided [33, 34]. Some studies select an arbitrary cut point without clear grouping basis and criteria in advance, which results in a decline in the predictive power of the model. Additionally, try not to temporarily convert continuous variables into categorical variables during the analysis stage, otherwise, internal validation and contraction regression coefficients should be conducted to adjust for overfitting [34]. Some studies directly exclude the inclusion of objects with missing data, and the methods for handling missing data have significant flaws. Missing data can negatively impact statistical analysis and model stability. For the processing of missing data, multiple interpolation or single interpolation methods can be employed [35]. Avoid relying on univariate analyses, where predictors are selected based on their statistical significance as a single predictor rather than in context with other predictors, which can lead to incorrect selection of predictors [36]. Combine expertise with practical analysis, not just statistical significance. Most articles fail to explain the complexity of the data. For complex data, provide a reasonable explanation or explain that the complexity of the data is not significant. Finally, issues such as model calibration, internal and external validation, overfitting, and underfitting should be taken into account.

The existing prediction model has some clinical significance. First, the predictors contained in these models may be candidate predictors for models to be developed in future studies. In addition, the usability of predictive models should be improved to make them more efficient in clinical use. Factors such as those that are difficult to measure and require additional scales or tools will increase the burden on users and should be minimized. Different locations, different institutions, and different users will have inconsistent validation results for model performance, and the risk of bias is high. More clinical studies should be conducted to verify the effectiveness of existing predictive models in reducing sarcopenia in dialysis patients. Finally, there are few studies on the clinical benefit evaluation of existing prediction models, which hinders the popularization and application of these models.

### Strength and limitations

First of all, our article is the first to focus on a systematic review of risk prediction models for sarcopenia in dialysis patients. Second, this study conducted an extensive literature search, and comprehensively screened the research in this field to reduce the possibility of missing research.

There are potential limitations to our study. First, we only included studies published in Chinese and English, so relevant studies in other languages may have been overlooked. Second, we limited our focus to the dialysis population and did not make predictions for the non-dialysis population in our systematic review. Third, Meta-analysis of predictive model studies could not be performed due to heterogeneity of data sources and methods. Finally, most of the models in this study lacked large samples and multi-centre external validation, which may have caused some bias in the results.

## Conclusion

In summary, our systematic review identified 13 studies describing 13 predictive models for sarcopenia in dialysis patients. There are a limited number of models for sarcopenia in dialysis patients of all ages. According to PROBAST, 12 included studies that developed or validated predictive models were evaluated as having a high risk of bias, one of which had an unknown risk. Current clinical models used to predict sarcopenia in dialysis patients do not meet PROBAST’s criteria. Researchers should learn and understand the PROBAST standard better before developing models. Future research should focus on validating and improving existing predictive models or developing new models with rigorous standards.

## Electronic supplementary material

Below is the link to the electronic supplementary material.

Supplementary Material 1

Supplementary Material 2

## Data Availability

No datasets were generated or analysed during the current study.
