# Supplementary material for: Prediction models for sarcopenia risk in dialysis patients: a systematic review and critical appraisal
Source: Aging Clin Exp Res. 2025 Jan 3;37(1):18. doi: 10.1007/s40520-024-02911-7 (PMC11698787; doi:10.1007/s40520-024-02911-7)
Supplement: Supplementary file 1 — Supplementary Material 1 [file 40520_2024_2911_MOESM1_ESM.docx]

**Supplemental Material**

**Table S1. Search strategies in each database---2024.3.9**

| **Database** | **Search strategies** | **Results** |
| --- | --- | --- |
| **PubMed** | #1 "Sarcopenia"[Mesh] ---9,915  #2 sarcopenia[Title/Abstract] OR sarcopenic[Title/Abstract] OR muscle mass[Title/Abstract] OR muscle strength[Title/Abstract] OR hand strength[Title/Abstract] OR grip strength[Title/Abstract] OR muscle atrophy[Title/Abstract] OR muscle wasting[Title/Abstract] ---87,799  #3 #1 OR #2---88,203  #4 prediction model[Title/Abstract] OR prediction[Title/Abstract] OR predict model[Title/Abstract] OR risk prediction[Title/Abstract] OR risk factors[Title/Abstract] OR risk assessment[Title/Abstract] OR prognostic model[Title/Abstract] OR model[Title/Abstract] OR nomogram[Title/Abstract]---3,630,944  #5 #3 AND #4---13,605 | 13,605 |
| **Web of Science** | #1 ((TS=(Sarcopenia)) OR TI=(sarcopenia OR sarcopenic OR muscle mass OR muscle strength OR hand strength OR grip strength OR muscle atrophy OR muscle wasting)) OR AB=(sarcopenia OR sarcopenic OR muscle mass OR muscle strength OR hand strength OR grip strength OR muscle atrophy OR muscle wasting)---227,995  #2 TI=(prediction model OR prediction OR predict model OR risk prediction OR risk factors OR risk assessment OR prognostic model OR model OR nomogram) OR AB=(prediction model OR prediction OR predict model OR risk prediction OR risk factors OR risk assessment OR prognostic model OR model OR nomogram)---10,567,826  #3 #1 AND #2---[48,701](https://webofscience.clarivate.cn/wos/alldb/summary/ce52af1e-387c-4f3e-8ad2-ad3b0194bf94-bd8093ba/relevance/1) | [48,701](https://webofscience.clarivate.cn/wos/alldb/summary/ce52af1e-387c-4f3e-8ad2-ad3b0194bf94-bd8093ba/relevance/1) |
| **Embase** | #1 'sarcopenia'/exp OR sarcopenia:ti,ab,kw OR sarcopenic:ti,ab,kw OR 'muscle mass':ti,ab,kw OR 'muscle strength':ti,ab,kw OR 'hand strength':ti,ab,kw OR 'grip strength':ti,ab,kw OR 'muscle atrophy':ti,ab,kw OR 'muscle wasting':ti,ab,kw---127,967  #2 'prediction model':ti,ab,kw OR prediction:ti,ab,kw OR 'predict model':ti,ab,kw OR 'risk prediction':ti,ab,kw OR 'risk factors':ti,ab,kw OR 'risk assessment':ti,ab,kw OR 'prognostic model':ti,ab,kw OR model:ti,ab,kw OR nomogram:ti,ab,kw---4,709,467  #3 #1 AND #2---20,937 | 20,937 |
| **Cochrane library** | #1 MeSH descriptor: [Sarcopenia] explode all trees---862  #2 (sarcopenia OR sarcopenic OR muscle mass OR muscle strength OR hand strength OR grip strength OR muscle atrophy OR muscle wasting):ti,ab,kw (Word variations have been searched)---43,522  #3 #1 OR #2---43,522  #4 (prediction model OR prediction OR predict model OR risk prediction OR risk factors OR risk assessment OR prognostic model OR model OR nomogram):ti,ab,kw (Word variations have been searched)---397,172  #5 #3 AND #4---8,611 | 8,611 |
| **CNKI** | #1 (SU=Sarcopenia) OR (TKA=sarcopenia OR sarcopenic OR muscle mass OR muscle strength OR hand strength OR grip strength OR muscle atrophy OR muscle wasting)---80,600  #2 (TKA=prediction model OR prediction OR predict model OR risk prediction OR risk factors OR risk assessment OR prognostic model OR model OR nomogram)---11,547,700  #3 #1 AND #2---12,600 | 12,600 |
